# Food Insecurity, Anemia and Vitamin A Deficiency in Brazilian Children Aged between 6 and 59 Months of Age: Brazilian National Survey on Child Nutrition (ENANI-2019)

**DOI:** 10.1016/j.cdnut.2025.104567

**Published:** 2025-02-17

**Authors:** Letícia Ramos da Silva, Paula Normando, Raquel Machado Schincaglia, Inês Rugani Ribeiro de Castro, Pedro Gomes Andrade, Talita Lelis Berti, Elisa Maria de Aquino Lacerda, Nadya Helena Alves-Santos, Letícia Barroso Vertulli Carneiro, Gilberto Kac

**Affiliations:** 1 Instituto de Nutrição Josué de Castro, Universidade Federal do Rio de Janeiro, Rio de Janeiro, Brazil; 2 Faculdade de Nutrição, Universidade Federal de Goiás, Goiás, Brazil; 3 Instituto de Nutrição, Universidade do Estado do Rio de Janeiro, Rio de Janeiro, Brazil; 4 Instituto de Pesquisa Econômica Aplicada, Rio de Janeiro, Brazil; 5 Instituto de Ciências da Saúde, Faculdade de Nutrição, Universidade Federal do Pará, Pará, Brazil; 6 Instituto de Estudos em Saúde Coletiva, Universidade Federal do Rio de Janeiro, Rio de Janeiro, Brazil

**Keywords:** food insecurity, micronutrients, preschool child, national survey, Brazil

## Abstract

**Background:**

Anemia and vitamin A deficiency (VAD) can be related to poverty and food insecurity (FI), which can increase risk of stunting and delayed child development.

**Objectives:**

This study aims to assess the association between FI and the occurrence of anemia and VAD in Brazilian children aged 6–59 months.

**Methods:**

Data from 6020 children from the Brazilian National Survey on Child Nutrition (2019) were used. FI was assessed using the Brazilian Food Insecurity Scale, classifying households into food security (FS) and FI levels. The outcomes were anemia [hemoglobin <10.5 g/dL (6–23 months) and hemoglobin <11 g/dL (24–59 months)] and VAD (retinol corrected by C-reactive protein <0.7 μmol/L). FI frequencies, anemia, and VAD prevalences were calculated according to FI levels, 95% confidence intervals (CIs), and *P*-trend. The adjusted prevalence ratio (PR) was estimated using quasi-Poisson regression.

**Results:**

The prevalence of mild, moderate, and severe FI was 37.7% (95% CI: 32.0%, 43.4%), 6.2% (95% CI: 4.8%, 7.6%), and 4.2% (95% CI: 3.1%, 5.3%), respectively. The prevalence of anemia was 7.1% (95% CI: 5.9%, 8.3%), and VAD was 3.0% (95% CI: 2.5%, 3.6%). A significant linear trend (*P*-trend < 0.001) was observed in the prevalence of anemia according to the degree of FI: severe (15.5%; 95% CI: 8.1%, 22.9%), moderate (10.0%; 95% CI: 5.4%, 14.6%), mild (6.6%; 95% CI: 4.9%, 8.3%), and FS (6.4%; 95% CI: 4.7%, 8.1%). Children living in households with severe FI had an 82% higher prevalence of anemia (PR: 1.82; 95% CI: 1.40, 4.17) than those living in FS. A significant linear trend (*P*-trend < 0.001) was observed in the prevalence of VAD according to the degree of FI: severe (3.3%; 95% CI: 0.0%, 6.7%), moderate (5.8%; 95% CI: 2.3%, 9.2%), FI (2.8%; 95% CI: 1.9%, 3.7%), and FS (2.9%; 95% CI: 2.1%, 3.7%). No association was observed between FI and VAD.

**Conclusions:**

Severe FI was associated with anemia among Brazilian children aged 6–59 months.

## Introduction

Anemia and vitamin A deficiency (VAD) are commonly prioritized nutritional problems on the global public health agenda because these nutritional deficiencies have a high prevalence in several countries [1,2]. These deficiencies affect mainly infants and preschoolers because of the considerable requirements for these nutrients and the greater biological vulnerability of this age group [3].

A study on the global burden of anemia estimated that the prevalence in children aged under 5 y was 41.4% in 2021 [4]. Estimates from population-based studies conducted in low- and middle-income countries revealed a reduction in the prevalence of VAD in children aged between 6 and 59 mo from 39% in 1991 to 29% in 2013 [5]. In Brazil, a comparison of data from the National Demographic and Health Survey (PNDS-2006) [6] and the Brazilian National Survey on Child Nutrition (ENANI-2019) [7] showed a reduction of 51.7% (from 20.9% to 10.1%) in the prevalence of anemia and 65.5% (from 17.4% to 6.0%, data not corrected for inflammation) in the prevalence of VAD.

Several socioeconomic factors, such as low-income, low education level of the caregiver, poor basic sanitation conditions, and lack of access to health services, have been associated with a higher prevalence of anemia and VAD [4,8]. In turn, these nutritional problems increase risk of stunting and delayed child development and are usually more prevalent in populations living in vulnerable conditions, in which food insecurity (FI) is common.

FI occurs when an individual does not have access to sufficient, safe, and nutritious food [9] and can be measured differently across countries, with variations in definitions, assessment tools, and criteria. In some countries, such as the United States, the Household Food Security Survey Module is used to assess FI based on the frequency of food access problems and the severity of hunger experienced [10]. In Brazil, FI is estimated using the Brazilian Household Food Insecurity Measurement Scale (EBIA), which categorizes FI into mild, moderate, and severe levels based on household access to food. Mild FI involves limited concerns about food access, leading to reduced meal variety or quality. Moderate FI results in skipped meals and occasional hunger, whereas severe FI causes frequent hunger and a significant lack of food [11,12]. These differences in definitions and assessment methods can lead to variations in identifying and understanding FI, highlighting the importance of considering cultural and contextual factors when comparing FI across nations [10].

From 2017 to 2018, the prevalence of FI in Brazilian households with children aged under 5 y was 48.6%, according to data from the Consumer Expenditure Survey [13], and 47.1%, according to data from the ENANI-2019 [14]. Conditions of FI suggest a decrease in the adequate supply of nutrients [15,16] and may increase risk of anemia and VAD in children [17]. FI in childhood also increases risk of growth faltering in children [18] and can negatively impact cognitive, motor, language, and socioemotional development [19].

Several studies in Brazil and worldwide have investigated the associations between FI and anemia [[20], [21], [22], [23], [24], [25], [26], [27], [28]] and between FI and VAD [22,[26], [27], [28], [29], [30]] in children. However, in Brazil, the only nationally representative study used data from 2006 [17], resulting in knowledge gaps. This study aimed to evaluate the association between FI and the occurrence of anemia and VAD in Brazilian children aged between 6 and 59 mo who participated in the ENANI-2019.

## Methods

### Study design

This manuscript uses data from the ENANI-2019, a nationally representative population-based household survey that investigated the nutritional profile of Brazilian children aged under 5 y. ENANI-2019 measured anthropometric nutritional status, micronutrient deficiency, and dietary practices. The survey adopted a complex probability sampling with stratification by macroregion (5 geographically and administratively defined macroregions closely related to the country's socioeconomic inequalities) and clustering analysis according to census tracts. The basic weights were adjusted through calibration to ensure the sample was representative. More details about the study design, population, treatment of nonresponses, and the study complex sample, including weighting, calibration, and poststratification, were described previously [[31], [32], [33], [34]].

### Data collection and participants

**FIGURE 1:**
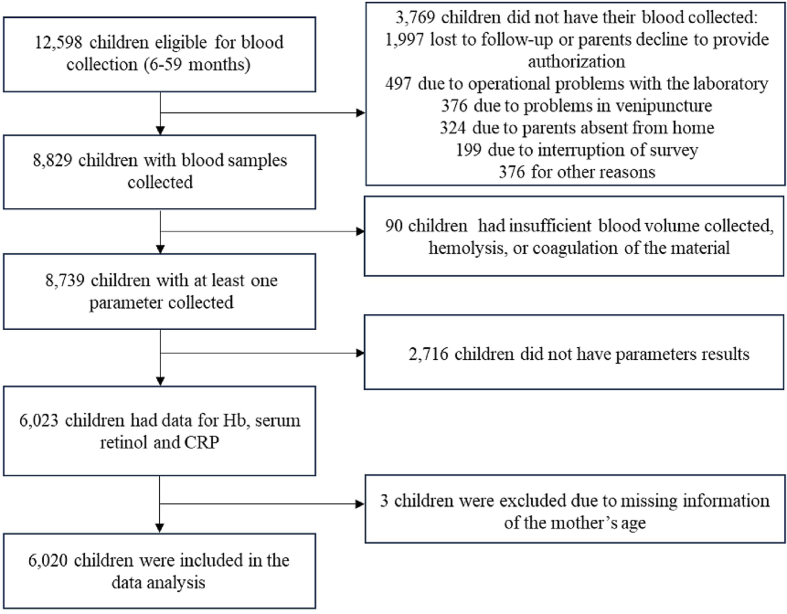
Data collection flowchart. Brazilian National Survey on Child Nutrition (ENANI-2019). CRP, C-reactive protein; Hb, hemoglobin.

The ENANI-2019 data collection took place between February 2019 and March 2020. The ENANI-2019 included a probability sample of 12,524 households and 14,558 children distributed in 123 municipalities of the 26 states of Brazil and the Federal District. Among these, 12,598 children aged 6–59 mo were considered eligible for blood sampling, and samples were collected successfully from 8829 children. Of these children, 8739 had available laboratory results; 2716 children were excluded because of missing results for blood parameters, such as retinol, hemoglobin (Hb), and C-reactive protein (CRP), considered as priorities in this study, resulting in a sample of 6023 children. In addition, 3 children were excluded because the age of the mother/caregiver was not provided. Therefore, the final sample consisted of 6020 children aged between 6 and 59 mo (Figure 1). Trained interviewers used a standardized questionnaire to collect sociodemographic and health data.

### Blood collection and laboratory tests

A total of 8 mL of blood was collected by venous puncture into EDTA (2 mL) and trace (6 mL) tubes. The trace tubes were wrapped in aluminum foil to avoid contact with light and ensure sample stability for serum retinol analysis. The serum retinol concentration was assessed by HPLC with ultraviolet detection (HPLC, Chromsystems). The complete blood count in whole blood was assessed using a hematological analyzer, with cell analysis performed using flow cytometry (UniCell DxH, Beckman Coulter). These methods are the reference standards for diagnosing anemia [35] and VAD [36]. CRP level was measured by immunoturbidimetry (AU5800, Beckman Coulter). More details about the blood collection and laboratory analysis procedures were described previously [37].

The outcomes of this study were anemia and VAD. Anemia was defined according to the cutoff point established by the WHO in 2024: Hb concentrations <10.5 g/dL for children aged 6–23 mo and <11 g/dL for children aged 24–59 mo [38]. For comparison, a complementary analysis using a cutoff point of Hb <11 g/dL [39] for children aged between 6 and 59 mo was performed. VAD was defined by a serum retinol concentration <0.7 μmol/L [2]. Serum retinol concentrations were adjusted using CRP data to correct the influence of inflammation, according to the approach recommended by the Biomarkers Reflecting Inflammation and Nutritional Determinants of Anemia (BRINDA) group [40] for preschool-aged children. Uncorrected complementary data are also presented.

### Assessment of household FI

FI was the exposure variable and was assessed using the EBIA. The EBIA was validated for the Brazilian population and is used to determine the perception and experience of hunger at the household level [11,12]. The EBIA that is administered to households with children aged under 18 y consists of 14 items that measure household accessibility to food in the 3 mo prior to the assessment. The answers are dichotomous (yes/no); 1 point is assigned to each affirmative answer, and 0 points are assigned to each negative answer. The total score is obtained by adding the positive responses to each of the 14 items. On the basis of this sum, the classification for food security (FS) and FI was made: FS (0 points); mild FI (between 1 and 5 points); moderate FI, 6–9 points; and severe FI, 10–14 points [11,12].

### Variables

The following variables were considered in the analysis: macroregion (North, Northeast, Southeast, South, or Midwest), household location (urban or rural), child's sex (female or male), child's age (6–23 or 24–59 mo, considering the age-specific Hb cutoffs to define anemia and on established developmental milestones and nutritional guidelines), skin color/race of the child reported by the mother/caregiver (White, mixed-race, or Black), skin color/race self-reported by the mother/caregiver (White, mixed-race, or Black), the total number of children per household (1, 2, or ≥3), mother/caregiver's age (<20, 20–34, or ≥35 y, considering the relationship between age, socioeconomic characteristics, biological maturity, and health), years of education of the mother/caregiver (0–7, 8–10, 11, or ≥12 y, classification aligned with the Brazilian education system), family income per capita [≤ ¼, ¼ to ½, ½ to 1, or >1 Brazilian minimum wage, in 2019: R$ 998.00 (US$ 268.28) and in 2020 R$ 1045.00 (US$ 213.17)], receiving a benefit from the Bolsa Família Program (BFP) (yes or no) and mother/caregiver living with a partner (yes or no). We also included variables related to access to basic urban infrastructure services and health and education services, such as type of sanitary sewage (general sewage or rainwater network, pit, or ditch), water supply (general network distribution system or well), access to garbage collection (yes or no), access to electricity (yes or no), enrollment in a day care center or school (day care center or public school, day care center or private school or none/never attended) and time spent in day care or school (full-time, part-time, or not enrolled).

### Statistical analysis

All analyses used calibrated sample weights because stratification, clustering, and unequal probabilities were used in the selection stages, resulting in a complex study sample. The frequencies and confidence intervals (95% CIs) of the variables of interest for the total sample were calculated, and the prevalence, prevalence ratio (PR), and 95% CIs for anemia and VAD were calculated according to FI level (mild, moderate, and severe). The coefficient of variation (CV) is a measure of precision. A CV < 30% indicates a good level of precision [41]; higher values should be interpreted with caution because they suggest that the sample is not large enough to allow an estimate at the population level. The Cochran‒Armitage test was also performed to verify the presence of ordered *P*-trends for the prevalence of anemia and VAD according to the levels of FS and FI.

Crude and adjusted quasi-Poisson regression models were used to assess the association between FI severity and anemia/VAD. The magnitude of the association was estimated using the PR with its corresponding 95% CI. The significance level adopted was 5%. Prevalence and prevalence ratios of anemia/VAD were also calculated for children in different age groups (6–23 mo, 24–59 mo, and 6–59 mo). These results are presented in Supplemental Tables.

We generated a directed acyclic graph (DAG) through the online tool DAGitty (//dagitty.net) to identify possible confounders. The DAG is a causal diagram model built based on a literature review that allows the creation of models of causal interactions and the identification of the minimum necessary adjustment. The following confounders were considered: cash transfer program (BFP), age of the child, per capita family income (minimum wage), macroregion, age of mother/caregiver, and mother/caregiver education level (Supplemental Figure 1A and B). We performed stratified analyses by age groups (6–23 and 24–59 mo) to explore potential differences in the prevalence of anemia and VAD across FI levels.

All analyses were performed using the survey package in R version 4.0.2 (www.r-project.org) in the collaborative environment of Jupyter*,* and the sample design of the ENANI-2019 was incorporated.

#### Ethical aspects

The ENANI-2019 was approved by the Research Ethics Committee of the Clementino Fraga Filho University Hospital of the Federal University of Rio de Janeiro and received a certificate of presentation for ethical review (process n. 89798718.7.0000.5257). Participation in the study was voluntary, and signed copies of the informed consent form were obtained.

## Results

The prevalence of FI was 48.1%, with 37.7% (95% CI: 32.0%, 43.4%) for mild FI, 6.2% (95% CI: 4.8%, 7.6%) for moderate FI, and 4.2% (95% CI: 3.1%, 5.3%) for severe FI. A total of 38% (95% CI: 34.9%, 41.6%) of the families received the BFP benefit, and 87.5% lived in households with an income ≤1 minimum wage. Most mothers or caregivers of the children lived with a partner (73.4%; 95% CI: 70.9%, 75.9%), were mixed race (57%; 95% CI: 53.1%, 60.9%), were aged between 20 and 34 y (67.7%; 95% CI: 64.9%, 70.4%) and had 11 or more years of schooling (53.8%) (Table 1).

**TABLE 1:** Characterization according to demographic and socioeconomic variables of Brazilian children aged between 6 and 59 mo [Brazilian National Survey on Child Nutrition (ENANI-2019)^1^].

Variables	Frequency (%)	95% CI	CV (%)^2^
Food security and levels of food insecurity			
Food security	51.9	45.4, 58.4	6.3
Mild food insecurity	37.7	32.0, 43.4	7.7
Moderate food insecurity	6.2	4.8, 7.6	11.6
Severe food insecurity	4.2	3.1, 5.3	13.7
Macroregion			
North	10.9	10.9, 10.9	0.0
Northeast	28.1	28.1, 28.1	0.0
Southeast	39.3	39.3, 39.3	0.0
South	13.4	13.4, 13.5	0.0
Midwest	8.3	8.3, 8.3	0.0
Household location			
Urban	96.9	95.1, 98.7	1.0
Rural	3.1	1.3, 4.9	30.1
Child's sex			
Male	51.2	51.2, 51.2	0.0
Female	48.8	48.8, 48.8	0. 0
Child's age (mo)			
6–23	34.9	34.3, 35.4	0.8
24–59	65.1	64.6, 65.7	0.4
Family income per capita (Brazilian minimum wage)^3^			
≤1/4	28.3	24.2, 32.3	7.4
1/4 a 1/2	35.6	32.5, 38.8	4.5
1/2 a 1	23.6	20.8, 26.5	6.1
>1	12.5	10.1, 14.9	9.8
Skin color/race of the child reported by the mother/caregiver^4^			
White	41.0	37.7, 44.4	4.2
Mixed race	52.0	48.7, 55.4	3.3
Black	6.4	5.0, 7.7	10.5
Skin color/race self-reported by the mother/caregiver^4^			
White	30.3	27.3, 33.3	5.0
Mixed race	57.0	53.1, 60.9	3.5
Black	11.5	9.3, 13.8	9.8
Mother/caregiver educational level (completed years)			
0–7	24.0	21.7, 26.4	5.0
8–10	22.2	19.9, 24.4	5.2
11	37.2	34.8, 39.6	3.3
≥12	16.6	14.6, 18.6	6.1
Mother/caregiver living with a partner			
Yes	73.4	70.9, 75.9	1.7
No	26.6	24.1, 29.1	4.7
Mother/caregiver's age (y)			
<20	6.7	5.3, 8.0	10.2
20–34	67.7	64.9, 70.4	2.1
≥35	25.7	23.2, 28.1	4.9
Receiving a benefit from the Bolsa Família Program			
Yes	38.3	34.9, 41.6	4.5
No	61.7	58.4, 65.1	2.8
Total number of children per household			
1	71.4	68.0, 74.9	2.5
2	26.0	23.0, 29.0	6.0
≥3	2.6	1.5, 3.7	21.5
Type of sewage system^5^			
General or rainwater system	72.4	68.8, 76.1	2.5
Pit^6^	24.8	21.2, 28.4	7.3
Ditch	1.7	0.4, 2.9	38.6
Water supply^7^			
General distribution system	93.6	91.2, 96.0	1.3
Well^8^	6.1	3.8, 8.5	19.6
Access to garbage collection			
Yes^9^	98.2	97.1, 99.3	0.6
No	1.8	0.7, 2.9	30.2
Access to electricity			
Yes	99.8	99.6, 100.1	0.1
No	0.2	0.0, 0.4	69.0
Enrollment in a day care center or school			
Day care center or public school	33.2	29.5, 36.9	5.7
Day care center or private school 10	10.5	8.6, 12.3	9.1
None/never attended^11^	56.3	52.6, 60.1	3.4
Time spent in day care or school			
Full-time	14.3	11.0, 17.5	11.7
Partial^12^	29.4	26.6, 32.3	4.9
None/never attended^11^	56.3	52.6, 60.1	3.4

Abbreviations: CI, confidence interval; CV, coefficient of variation.

1 To perform this characterization, only the ENANI-2019 database of specific micronutrients was used, not the general database of the study.

2 The coefficient of variation is a measure of dispersion that indicates the heterogeneity of the data. Values >30% are considered imprecise.

3 Family income was measured according to the Brazilian minimum wage of the period in which the survey took place, in 2019: R$ 998.00 (US$ 268.28) and in 2020 R$ 1045.00 (US$ 213.17).

4 People of Asian race/color and Indigenous people were omitted from the results due to the low precision of the estimates and because they represent <1% of the sample.

5 The category “others,” which includes direct access to rivers, lakes, or seas, had its results omitted because they represent <2% of the sample.

6 Septic tank or rudimentary tank.

7 The category “others,” which includes water trucks, rainwater stored in cisterns, rainwater stored in other ways, rivers, ponds, lakes, and streams, had its results omitted due to the low precision of the estimates and because it represents <1% of the sample.

8 Well or spring on the property or outside the property.

9 Collected directly by a cleaning service or in a cleaning service dumpster.

10 Including day care centers and schools maintained by churches.

11 No, never attended, not applicable, or other.

12 Only in the morning or only in the afternoon.

The prevalence of anemia showed a significant linear trend according to FI severity: severe, 15.5% (95% CI: 8.1%, 22.9%); moderate, 10.0% (95% CI: 5.4%, 14.6%); mild, 6.6% (95% CI: 4.9%, 8.3%); and FS, 6.4% (95% CI: 4.7%, 8.1%) (*P*-trend <0.001). The prevalence of VAD also showed a significant linear trend according to FI severity: severe, 3.3% (95% CI: 0.0%, 6.7%); moderate, 5.8% (95% CI: 2.3%, 9.2%); mild, 2.8% (95% CI: 1.9%, 3.7%); and FS, 2.9% (95% CI: 2.1%, 3.7%) (*P*-trend < 0.001) (Table 2).

**TABLE 2:** Prevalence of anemia and vitamin A deficiency according to food security and insecurity levels in Brazilian children aged 6–59 mo [Brazilian National Survey on Child Nutrition (ENANI-2019)^1^].

Variables	Prevalence (%)	Anemia^2^			Prevalence (%)	Vitamin A deficiency^3^		
		95% CI	CV (%)^4^	*P*-trend^5^		95% CI	CV (%)^4^	*P*-trend^5^
Brazil	7.1	5.9, 8.3	8.9		3.0	2.5, 3.6	9.0	
Level of food insecurity				<0.001				<0.001
Food security	6.4	4.7, 8.1	13.7		2.9	2.1, 3.7	13.8	
Mild food insecurity	6.6	4.9, 8.3	12.9		2.8	1.9, 3.7	16.7	
Moderate food insecurity	10.0	5.4, 14.6	23.3		5.8	2.3, 9.2	30.9	
Severe food insecurity	15.5	8.1, 22.9	24.2		3.3	0.0, 6.7	51.6	

Abbreviations: BRINDA, Biomarkers Reflecting Inflammation and Nutritional Determinants of Anemia; CI, confidence interval; CV, coefficient of variation; Hb, hemoglobin.

1 To perform this characterization, only the ENANI-2019 database of specific micronutrients was used, not the general study database.

2 Cutoff point for anemia classification: Hb concentration <10.5 g/dL between 6 and 23 mo and Hb < 11.0 g/dL between 24 and 59 mo.

3 Cutoff point for vitamin A deficiency classification: serum retinol <0.70 μmol/L. Vitamin A deficiency was adjusted according to the BRINDA method [37].

4 Coefficient of variation is a measure of dispersion that indicates data heterogeneity. Values >30% are considered imprecise.

5 The *P*-trend was performed using the Cochran Armitage test.

The prevalence of anemia was 82% greater in children living in households with severe FI than in those living in households with FS (PR: 1.82; 95% CI: 1.06, 3.15) (Table 3). No associations were found between FI and the prevalence of VAD (Table 4).

**TABLE 3:** Prevalence ratio between FI and anemia in Brazilian children aged 6–59 mo [Brazilian National Survey on Child Nutrition (ENANI-2019)^1^].

Variables	Unadjusted model			Adjusted model^2^		
	PR	95% CI	*P* value	PR	95% CI	*P* value
Level of food insecurity						
Food security (reference)	1.00			1.00		
Mild food insecurity	1.03	0.72, 1.47	0.859	0.90	0.63, 1.28	0.563
Moderate food insecurity	1.56	0.93, 2.62	0.089	1.01	0,59, 1.75	0.945
Severe food insecurity	2.42	1.40, 4.17	0.001	1.82	1.06, 3.15	0.029

PR estimates were considered statistically significant when *P* <0.05. The analyses were performed using quasi-Poisson regression models for the independent variable and anemia. Cutoff point for anemia classification: Hb concentration <10.5 g/dL between 6 and 23 mo and Hb <11.0 g/dL between 24 and 59 mo.

Abbreviations: CI, confidence interval; FI, food insecurity; Hb, hemoglobin; PR, prevalence ratio.

1 To perform this characterization, only the ENANI-2019 database of specific micronutrients was used, not the general study database.

2 Model adjusted for the following confounding factors identified through directed acyclic graphics: income transfer program (receipt of Bolsa Família Program benefit), child’s age, per capita family income (minimum wage), macroregion, maternal/caregiver age, and maternal/caregiver education level.

**TABLE 4:** Prevalence ratio between FI and VAD in Brazilian children aged 6–59 mo [Brazilian National Survey on Child Nutrition (ENANI-2019)^1^].

Variables	Unadjusted model			Adjusted model^2^		
	PR	95% CI	*P* value	PR	95% CI	*P* value
Level of food insecurity						
Food security (reference)	1.00			1.00		
Mild food insecurity	0.95	0.59, 1.55	0.859	0.83	0.51, 1.35	0.474
Moderate food insecurity	1.99	1.02, 3.87	0.041	1.45	0.69, 3.03	0.323
Severe food insecurity	1.15	0.41, 3.20	0.782	0.84	0.28, 2.48	0.759

PR estimates were considered statistically significant when *P* <0.05. The analyses were performed using quasi-Poisson regression models for the independent variable and anemia. Cutoff point for VAD: serum retinol <0.70 μmol/L. VAD was adjusted according to the BRINDA method.

Abbreviations: BRINDA, Biomarkers Reflecting Inflammation and Nutritional Determinants of Anemia; CI, confidence interval; FI, food insecurity; PR, prevalence ratio; VAD, vitamin A deficiency.

1 To perform this characterization, only the ENANI-2019 database of specific micronutrients was used, not the general study database.

2 Model adjusted for the following confounding factors identified through directed acyclic graphics: income transfer program (receipt of Bolsa Família Program benefit), child’s age, per capita family income (minimum wage), macroregion, maternal/caregiver age, and maternal/caregiver education level.

The prevalence of anemia according to the 2001 WHO cutoff values and VAD without correction for inflammation were similar to those of anemia according to the 2024 WHO cutoff values and VAD corrected for inflammation (Supplemental Table 1). The absence of an association between FI and VAD remained when serum retinol without correction for CRP was used (PR: 0.71; 95% CI: 0.31, 1.63) (Supplemental Table 2).

In children who experienced severe FI, the prevalence of anemia in children aged 6–23 mo (30.8%; 95% CI: 15.2%, 46.3%) was greater than that in children aged 24–59 mo (10.6%; 95% CI: 2.9%, 18.4); however, no statistically significant differences were observed (Supplemental Table 3). No associations were observed between overall FI and anemia stratified by age into 6–23 mo and 24–59 mo (Supplemental Table 4).

## Discussion

The results of this study utilizing data from the ENANI-2019 revealed a prevalence of anemia of 7.1% according to the 2024 WHO Hb cutoff values [38] and 9.8% according to the 2001 cutoff values [39]. The prevalence of VAD was 3.0% when corrected for inflammation using CRP level and 5.7% without correction. These results indicate that anemia and VAD represent a public health problem among Brazilian children [2,38]. The prevalence of FS and mild, moderate, and severe FI were 51.9%, 37.7%, 6.2%, and 4.2%, respectively. FI was not a risk factor for a higher prevalence of VAD. In contrast, the prevalence of anemia was 15.5% in children experiencing severe FI, indicating that these children had an 82% greater risk of developing anemia than those with FS after controlling for confounders. Thus, the ENANI-2019 is the first national population-based study to show an association between severe FI and anemia.

Between 2004 and 2013, Brazil significantly improved food and nutrition security (FNS) because of the favorable economic and political environment and the adoption of public policies to promote FNS [42]. During this period, the National Household Sample Survey (PNAD) indicated that the prevalence of FI in households with children aged under 5 y decreased from 47.4% (PNAD-2004) to 41.2% (PNAD-2009) to 32.4% (PNAD-2013) [43]. From 2016 onward, the public policies promoting FNS were dismantled, contributing to a setback in this field [44]. The 48.1% prevalence of FI observed by the ENANI-2019 should be discussed in this context. In 2023, the continuous PNAD results revealed a prevalence of FI of 36.4% in households with children aged under 5 y [43]; this new reduction can be partially explained as a consequence of the redirection of public actions and policies aimed at addressing structural concerns and overcoming historical inequalities, in addition to combating the problems caused by the COVID-19 pandemic.

The trends in FI prevalence are influenced by economic cycles and public policies of centralization and decentralization in the fight against hunger and poverty. Among these policies, we highlight the National School Feeding Program, which guarantees that children enrolled in day care centers or public schools in Brazil receive an adequate and healthy diet while they are there, and the BFP, a Brazilian income transfer program that supports families in socially vulnerable situations, especially those with children [44].

A diet with poor food and nutrition quality, as observed in low- and middle-income countries, may reflect social disparities and highlight the presence of FI in the context of poverty and other social vulnerabilities in children. FI can compromise the food supply and nutrients, resulting in a lower intake of essential nutrients, such as iron and vitamin A. Children living with FI may be more prone to developing a deficiency of these nutrients [45], which is common among children in middle- and low-income countries [2,38].

The prevalence of anemia (7.1%) and VAD (3.0% corrected for inflammation) observed in the present study differed from the prevalence identified by Gubert et al. [17] using data from the PNDS-2006 (anemia: 20.9% and VAD: 17.4%). The reduction in these prevalence rates can be partially attributed to the success of public policies such as the National Micronutrient Supplementation Program and food fortification programs [46]. A significant improvement in living conditions, the expansion of public health and food and nutrition policies implemented until 2015, and the resulting nutritional transition observed in Brazil, can also explain the reduction in the prevalence of anemia and VAD among children aged under 5 y.

The prevalence of anemia, according to the ENANI-2019, was 15.5% for children who experienced severe FI, which was 82% higher than that among children with FS. Using data from the PNDS-2006, Gubert et al. [17] reported no association between FI and anemia in children aged under 5 y. However, comparisons between PNDS-2006 and ENANI-2019 should be performed with caution because these surveys have important methodological differences, such as the different proportions of urban sectors (83% compared with 96.9%, respectively) and rural sectors (17% compared with 3.1%, respectively) surveyed [6] and the methods used to diagnose anemia and VAD (dry gout compared with venous puncture). Furthermore, although Gubert et al. [17] used 3 categories to explore this association (FS, mild and moderate FI, and severe FI), the present study included 4 categories (FS, mild FI, moderate FI, and severe FI) without grouping. Our results also differ from those of local studies in Southeast [25,26] and Northeast [27,28] Brazil, where no association between FI and anemia was observed. However, the ENANI-2019 data were representative of the child population, whereas the previous studies were conducted in restricted geographical and socioeconomic contexts.

The association between severe FI and anemia found in our study was similar to that reported in a population-based study conducted in Mexico with children aged between 12 and 59 mo [20]. However, our results differ from those of a study conducted in Colombia with schoolchildren aged between 5 and 12 y [22], which can be explained at least partially by the differences in age groups between the 2 studies. Our results also differ from those reported by Magaña et al. [21] in Mexico in children younger than 18 mo. These differences can be attributed to the fact that the families in the Mexican sample were beneficiaries of 2 social programs that contributed to improving diet quality, whereas, in the ENANI-2019, only 38.3% of families received a contribution from the BFP. Our results also differ from those observed in local studies conducted in Bangladesh [24] and Cambodia [23], in which no associations were found between FI and anemia.

The results of the literature concerning the association between FI and VAD are conflicting. The absence of an association between FI and VAD in the present study is consistent with the results of previous studies [17,22,[27], [28], [29]] performed in children in Brazil and Colombia. However, Chitekwe et al. [30] reported a significantly greater probability of VAD in Nepalese children living in households with severe FI than in those living in households with FS. Carneiro et al. [26] reported an inverse association between mild FI and retinol concentrations in a study conducted in Rio de Janeiro, Brazil.

The absence of an association between FI and VAD in the present study may be explained by the low prevalence of VAD in the study population (5.7% uncorrected, 3.0% after correction for inflammation). The most immediate cause of VAD is directly related to the ingestion of food sources. The average cost of a diet rich in iron (liver, red meat, chicken, and fish), the main micronutrient involved in anemia, is usually greater than the cost of food sources rich in vitamin A (spinach, kale, pumpkin, carrot, papaya, and mango). Thus, children with FI may have greater access to foods that are rich in vitamin A than to foods that are sources of iron. Although dark green vegetables are also sources of iron, the predominant form of iron in these vegetables has the lowest bioavailability for physiological functions, including Hb synthesis [45]. Among the children studied, vitamin A supplements were used in 35.2%, and iron supplements were used in 21.7% [47].

The metabolism of these nutrients may also explain the differences observed in the associations among FI, anemia, and VAD. Iron storage is strongly influenced by age, sex, and body size [48]. Iron deficiency occurs mainly in children because of inadequate complementary feeding and high growth and development velocity at this stage, which increases the demand for iron [18,19,48]. Vitamin A is fat-soluble and can be stored in large amounts in the liver and adipose tissue [49,50]. Healthy and well-nourished individuals who have eaten sufficient amounts of food and accumulated reserves of vitamin A may live for several weeks or even months before suffering the adverse effects of a diet deficient in vitamin A [48].

Notably, ENANI-2019 used a version of the EBIA with small changes in the wording of some questions, and the version was not designed and validated for the Brazilian population. However, to ensure the predictive ability of the FI results in the ENANI-2019, analyses were performed to verify the internal validity of the scale (Cronbach’s α coefficient*,* item response theory, likelihood ratio test, principal components, and Rasch model). Our study has some limitations, such as the reduced number of participants from rural areas and the lack of biomarker data in 2.806 infants because the blood sample volume collected was insufficient to perform all laboratory analyses. This later limitation was dealt with adjustments to children's baseline weights to compensate for nonresponse to biomarker data. Although the topic has already been discussed in the literature, 1 strength of this study is the representativeness of the study sample across Brazil regarding age group, sex, and macroregions. In addition, the method of blood collection and laboratory examinations used were considered the reference standard methods. Finally, another strength was the correction of the VAD estimates performed according to the method proposed by BRINDA [40], which recommends adjustments according to inflammation markers, such as CRP, in studies with preschool children.

In conclusion, our study revealed that, in 2019, 48.1% of Brazilian children aged between 6 and 59 mo lived in households with some degree of FI. In addition, Brazilian children who experienced severe FI were more likely to have anemia than were children with FS. The results of the ENANI-2019 also revealed that exposure to FI did not increase risk of VAD.

## Author contributions

The authors’ responsibilities were as follows – LRdS, PN, RMS, IRRdC, GK: contributed to the conception and design of the study; LRdS, RMS, PGA, TLB: contributed to the data processing and analysis; LRdS, PN, RMS, GK: contributed to the interpretation of the data; LRdS, PN, GK: contributed to the writing and critical article review; and all authors: reviewed, read, and approved the final manuscript.

## Data availability

Data described in the manuscript, code book, and analytic code will be made publicly and freely available without restriction at: https://enani.nutricao.ufrj.br/material-instrutivo-e-banco-de-dados/.

## Funding

This research received no external funding.

## Conflict of interest

The authors declare that they have no known competing financial interests or personal relationships that could have appeared to influence the work reported in this paper.
