# Supplementary material for: Food Insecurity, Anemia and Vitamin A Deficiency in Brazilian Children Aged between 6 and 59 Months of Age: Brazilian National Survey on Child Nutrition (ENANI-2019)
Source: Curr Dev Nutr. 2025 Feb 17;9(3):104567. doi: 10.1016/j.cdnut.2025.104567 (PMC11957489; doi:10.1016/j.cdnut.2025.104567)
Supplement: multimedia component 1 [file mmc1.pdf]

TITLE: Food insecurity, anemia and vitamin A deficiency in Brazilian children between 6 and 59 months of age: Brazilian National Survey on Child Nutrition (ENANI-2019)

AUTHOR: Letícia Ramos da Silva

Supplementary Figure1. Directed Acyclic Graph: (A) food insecurity and anemia (B) food insecurity and vitamin A.

Notes: The same minimum adjustments were suggested for supplementary figure 1 (A) and (B): income transfer program (receipt of benefit from the Bolsa Família program), child's age, per capita family income (minimum wage), macroregion, maternal/caregiver age and maternal/caregiver education level.

A

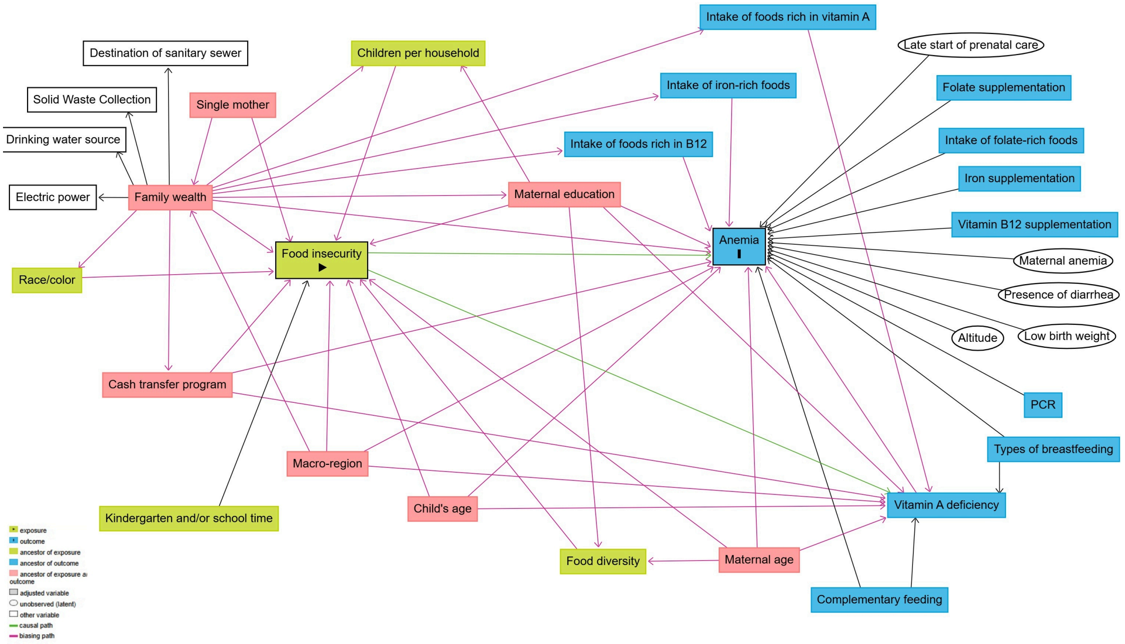

B

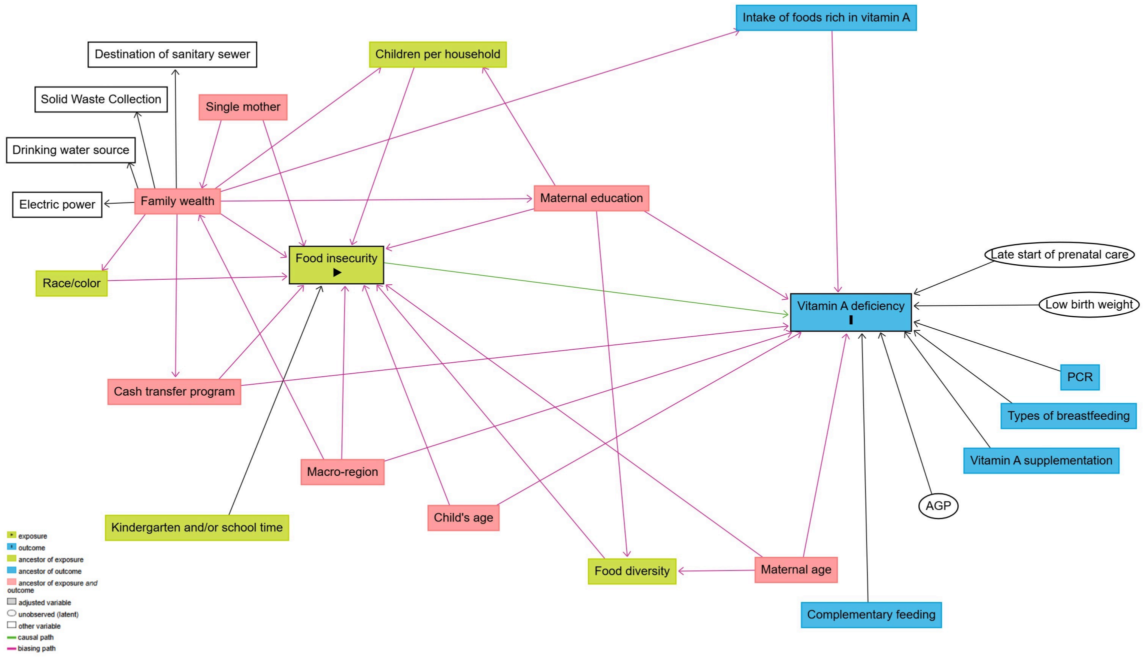

Supplementary table 1. Prevalence of anemia according to WHO 2001 cutoff, and vitamin A deficiency without correction for inflammation according to food security and insecurity levels, in Brazilian children between 6 and 59 months. Brazilian National Survey on Child Nutrition (ENANI-2019)[1]

Variables	Anemia[2]			Vitamin A deficiency[3]		
	Prevalence (%)	95% CI	CV (%)[4]	Prevalence (%)	95% CI	CV (%)[4]
Brazil	9.8	8.4; 11.2	7.5	5.7	4.9; 6.5	7.4
**Level of food insecurity**						
Food security	8.6	6.5; 10.6	12.1	5.3	4.3; 6.3	9.8
Mild food insecurity	10.2	8.0; 12.4	11.1	5.3	3.7; 7.0	16.0
Moderate food insecurity	12.7	7.2; 18.2	22.1	12.2	6.5; 18.0	23.9
Severe food insecurity	17.3	9.7; 24.8	22.4	4.8[e]	1.2; 8.4	37.9

Notes: 95%CI: confidence interval; CV: coefficient of variation.
[1]To perform this characterization, only the ENANI-2019 database of specific micronutrients was used, not the general study database.
[2]Cutoff point for anemia classification: Hb concentration < 11 g/dL in children between 6 and 59 months of age.
[3]Cutoff point for vitamin A deficiency classification: serum retinol < 0.70 µmol/L without correction for inflammation.
[4]Coefficient of variation is a measure of dispersion that indicates the heterogeneity of the data. Values > 30% are considered imprecise.

Supplementary table 2. Prevalence ratio between anemia with WHO 2001cutoff, and food insecurity and vitamin A deficiency without correction for inflammation and food insecurity in Brazilian children between 6 and 59 months. Brazilian National Survey on Child Nutrition (ENANI-2019)[1]

Variables	Adjusted model					
	**Anemia[2]**			**Vitamin A deficiency[3]**		
	**PR**	**95% CI**	**P-value**	**PR**	**95% CI**	**P-value**
**Level of food insecurity**						
Food security (reference)	1.00			1.00		
Mild food insecurity	1.18	0.86; 1.63	0.288	0.92	0.65; 1.31	0.661
Moderate food insecurity	1.47	0.89; 2.43	0.126	1.84	1.01; 3.36	0.044
Severe food insecurity	**2.01**	**1.22; 3.29**	**0.005**	0.71	0.31; 1.63	0.430

Notes: PR: Prevalence ratio; 95%CI: confidence interval; PR estimates were considered statistically significant when p-value < 0.05.
[1]To perform this characterization, only the ENANI-2019 database of specific micronutrients was used, not the general study database.
[2]Cutoff point for anemia classification: Hb concentration < 11 g/dL in children between 6 and 59 months of age.
[3]Cutoff point for vitamin A deficiency classification: serum retinol < 0.70 µmol/L without correction for inflammation.
Model adjusted for the following confounding factors identified through Directed Acyclic Graphics (DAG): income transfer program (receipt of Bolsa Família program benefit), child's age, per capita family income (minimum wage), macroregion, maternal/caregiver age, and maternal/caregiver education level.

Supplementary table 3. Prevalence of anemia according to age group in Brazilian children. Brazilian National Survey on Child Nutrition (ENANI-2019)[1]

Variables	6-23 months[2]			24-59 months[2]			6-59 months[2]		
	Prevalence (%)	95% CI	CV (%)[3]	Prevalence (%)	95% CI	CV (%)[3]	Prevalence (%)	95% CI	CV (%)[3]
Brazil	11.7	9.0; 14.4	11.8	4.8	3.5; 6.0	13.2	7.1	5.9; 8.3	8.9
**Level of food insecurity**									
Food security	12.0	8.3; 15.7	15.7	3.4	2.1; 4.7	19.0	6.4	4.7; 8.1	13.7
Mild food insecurity	9.5	6.2; 12.8	17.8	5.3	3.4; 7.1	18.1	6.6	4.9; 8.3	12.9
Moderate food insecurity	12.7	2.8; 22.6	39.6	8.4	4.4; 12.4	24.3	10.0	5.4; 14.6	23.3
Severe food insecurity	30.8	15.2; 46.3	25.7	10.6	2.9; 18.4	37.0	15.5	8.1; 22.9	24.2

Notes: 95%CI: confidence interval; CV: coefficient of variation.
[1]To perform this characterization, only the ENANI-2019 database of specific micronutrients was used, not the general study database.
[2]Cutoff point for anemia classification: Hb concentration < 10.5 g/dL between 6 and 23 months and Hb < 11.0 g/dL between 24 and 59 months.
[3]Coefficient of variation is a measure of dispersion that indicates data heterogeneity. Values > 30% are considered imprecise.

Supplementary table 4. Prevalence ratio between food insecurity and anemia according to age group in Brazilian children. Brazilian National Survey on Child Nutrition (ENANI-2019)[1]

**Variables**	**6-23 months**			**24-59 months**			**6-59 months**		
	**PR[2]**	**95% CI**	**P-value**	**PR[2]**	**95% CI**	**P-value**	**PR[2]**	**95% CI**	**P-value**
**Level of food insecurity**									
Food security (reference)	1.00			1.00			1.00		
Mild food insecurity	0.74	0.47; 1.15	0.186	1.19	0.68; 2.10	0.526	0.90	0.63; 1.28	0.563
Moderate food insecurity	0.89	0.42; 1.88	0.761	1.26	0.63; 2.50	0.499	1.01	0.59; 1.75	0.945
Severe food insecurity	1.74	0.87; 3.45	0.113	1.90	0.79; 4.57	0.150	**1.82**	**1.06; 3.15**	**0.029**

Notes: PR: Prevalence ratio; 95%CI: confidence interval; PR estimates were considered statistically significant when p-value < 0.05.

[1]To perform this characterization, only the ENANI-2019 database of specific micronutrients was used, not the general study database.

[2]Models adjusted for the following confounding factors identified through Directed Acyclic Graphics (DAG): income transfer program (receipt of Bolsa Família program benefit), child's age, per capita family income (minimum wage), macroregion, maternal/caregiver age, and maternal/caregiver education level.
